# Brain HIV-1 latently-infected reservoirs targeted by the suicide gene strategy

**DOI:** 10.1186/s12985-021-01584-2

**Published:** 2021-05-31

**Authors:** Sepideh Saeb, Mehrdad Ravanshad, Mahmoud Reza Pourkarim, Fadoua Daouad, Kazem Baesi, Olivier Rohr, Clémentine Wallet, Christian Schwartz

**Affiliations:** 1 Department of Virology, Faculty of Medical Sciences, Tarbiat Modares University, Tehran, Iran; 2 University of Strasbourg, Research Unit 7292, DHPI, IUT Louis Pasteur, Schiltigheim, France; 3 KU Leuven, Department of Microbiology, Immunology and Transplantation, Division of Clinical and Epidemiological Virology, 3000 Leuven, Belgium; 4 Hepatitis and AIDS Department, Pasteur Institute of Iran, Tehran, Iran

**Keywords:** HIV-1, Microglial, Latent reservoirs, Suicide gene

## Abstract

Reducing the pool of HIV-1 reservoirs in patients is a must to achieve functional cure. The most prominent HIV-1 cell reservoirs are resting CD4 + T cells and brain derived microglial cells. Infected microglial cells are believed to be the source of peripheral tissues reseedings and the emergence of drug resistance. Clearing infected cells from the brain is therefore crucial. However, many characteristics of microglial cells and the central nervous system make extremely difficult their eradication from brain reservoirs. Current methods, such as the “shock and kill”, the “block and lock” and gene editing strategies cannot override these difficulties. Therefore, new strategies have to be designed when considering the elimination of brain reservoirs. We set up an original gene suicide strategy using latently infected microglial cells as model cells. In this paper we provide proof of concept of this strategy.

## Introduction

Since the introduction of the combination antiretroviral therapy (cART) in 1996 the lethal HIV-1 infection has evolved to become a chronic disease. However, despite continual efforts definitive cure has not been achieved principally due to the existence of latently-infected HIV-1 reservoirs. Resting CD4 + T cells are known to be the principal reservoir of HIV but we have now abundant proofs that many other cell reservoirs such as hematopoietic stem cells, dendritic cells, microglial cells and cells from the monocyte-macrophage lineage (reviewed in [1–3]) exist. Furthermore, some of these reservoirs are found in sanctuaries such as the genital tract, the adipose tissue, the bone marrow or in the brain.

A prerequisite to successfully purge reservoirs is to understand the molecular mechanisms involved in the establishment and maintenance of HIV-1 latency. Understanding these mechanisms could help to identify new target proteins in the viral cycle, which are not affected by cART [4]. Some of these molecular mechanisms are known. We have notably shown that the cellular cofactor CTIP2 (Bcl11b) supports both the establishment and the maintenance of HIV-1 post-integration latency in microglial cells [5–7]. CTIP2 works as a scaffold protein by recruiting at least two different complexes in microglial cells. As part of a chromatin remodelling complex CTIP2 is associated with the lysine demethylase LSD1, the histone deacetylases HDAC1 and HDAC2, and the histone methyltransferase SUV39H1[5, 8–10]. CTIP2 is also involved in the control of the elongation process of gene transcription by inhibiting P-TEFb functions. [11, 12].

To date, three strategies are considered to eliminate reservoirs: 1) the “Shock and Kill”, 2) the alternative “Block and Lock” and 3) gene therapy including the gene editing based strategy. The “Shock and Kill” strategy is based on the reactivation of the latent virus followed by the intensification of cART, which eliminate reactivated viruses. This strategy seeks to achieve clearance of the reservoirs either by a cytopathic effect on the reactivated virus or inducing the immune system via the actions of cytotoxic T cells (CTLs) [13]. The “Shock and Kill” strategy today is only conceivable with circulating reservoirs such as the resting T CD4 + cells. Indeed, targeting the brain reservoirs with the “Shock and Kill” strategy is not feasible due to several characteristics of the central nervous system (CNS):

i. The CNS has barriers (the Brain Blood Barrier and the choroid plexus) which lower the penetration of drugs into the brain [14].
ii. The main cellular targets of cART are CNS resident macrophages including perivascular macrophages and microglial cells. However, few drugs are able to target the monocyte-macrophages lineage and microglial cells [15].
iii. Reactivation of the virus with Latency Reversing Agents (LRAs) leads to the synthesis of neurotoxic viral proteins such as Tat, Vpr and the gp120. Furthermore, reactivation of the virus activates macrophage/microglial cells and it is often correlated with CNS inflammation [16, 17].

An alternative approach to achieve long term control of HIV-1 in the absence of cART is to induce long lasting inhibition of HIV-1 gene expression [18]. Molecules inhibiting HIV-1 expression, called latency-promoting agents (LPAs), induce deep latency state (the Block) and prevent HIV-1 gene transcription (the Lock) [19]. Among LPAs, didehydro-corticostatin (dCA), an inhibitor of the transactivor Tat, had promising effect. However, a recent in vitro study described virus resistance to this agent [20]. Recently introduced gene therapies show promising results as well. The gene therapy targeting CCR5 co-receptor aims to replace wild type receptor by bone marrow transplantation of stem cells harboring CCR5 deletion mutation [21]. Stem-cells carrying a CCR5 ∆32 mutation confers resistance to HIV infection.

Mutational inactivation of HIV-1 by the gene editing CRISPR/Cas9 technique to disrupt the HIV genome is also promising [22]. Unfortunately, a major off-target of the gene editing strategy is the rise of unwanted gene mutations and chromosomal translocations [23]. Indeed, preliminary results of gene editing trials using CRISPR/Cas9 technique [24] showed that the virus was able to subvert the DNA repair machinery to rapidly evolve into CRISPR/Cas9 resistant strains [25–28]. In order to circumvent the emergence of resistant strains a variant of the CRISPR/Cas9 technology was developed inducing reactivation or deep latency in latently infected cells [29]. The method makes use of a defective Cas9 (dCas9) protein fused to virus activators or repressors in view of specific reactivation of latently-infected cells or suppressing HIV-1 expression, respectively [29] [30]. These experiments showed reactivation of HIV expression in CD4 + T cells and in microglial cell lines [31]. Remarkably, CRISPR/dCas9 when associated with HDAC inhibitors and PKC activators reactivated HIV in a synergistic manner [32].

Despite of considerable progress in new technologies in the field of gene therapy the purge of brain reservoirs (e.g. microglial cells) is still challenging. As evoked above and in [7, 33] HIV-1 brain reservoirs are sanctuaries in which cART does not work efficiently and new technics are needed to overcome limitations.

An original gene therapy has been introduced recently, which relies on the so called suicide gene therapy already well studied in the field of cancer [34, 35]. Indeed, this approach has been used in in vitro experiments to inhibit HIV-1 replication and proved to be safe and effective [36, 37]. Briefly, expression of the herpes simplex virus thymidine kinase is induced, which in turn converts the prodrug Ganciclovir into a toxic drug followed by apoptosis. We redesigned this strategy in order to specifically target latent brain reservoirs. A recombinant AAV vector including a Long Terminal Repeat-Thymidine kinase (LTR-TK) gene has been developed. The idea was that the reactivated virus produces the trans-activator Tat in latently-infected reservoirs, which in turn drives the expression of the thymidine kinase by binding the HIV-1 promoter LTR. In the presence of the prodrug Ganciclovir, we thus expected to induce apoptosis preferentially in reactivated cells. We used specifically the AAV vector to target latently-infected reservoirs, which is nonpathogenic for human and has the ability to infect both dividing and non-dividing cells [38]. Another advantage is that the vector exhibits stable transgene expression with low risk of insertional mutagenesis [38]. Last but not least, some AAV serotypes have been shown to infect cells in the brain following intravascular administration in preclinical trials [39, 40]. We show in this paper that a model of latently infected microglial cell when reactivated by Romidepsin and transduced by a recombinant AAV becomes apoptotic.

## Materials and methods

### AAV production

#### Design of the recombinant AAV viral vector

**Fig. 1:**
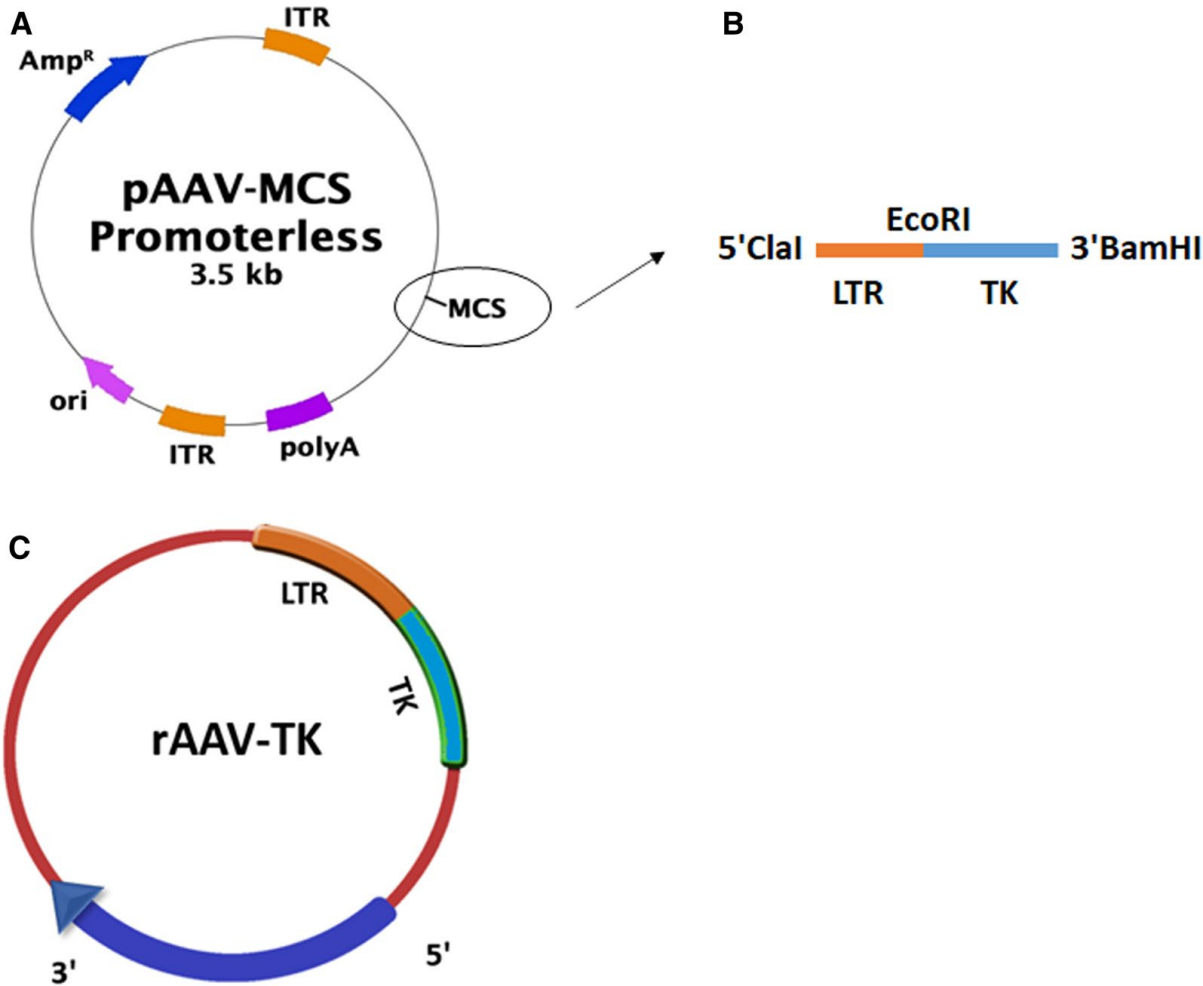
Cloning strategy for making rAAV-TK vector. HIV-1 LTR sequence was cloned in multiple cloning site of pAAV-MCS promoterless expression vector (**a**), between ClaI and EcoRI restriction enzyme sites. The coding region of TK gene was also incorporated into this vector at the downstream of LTR; between EcoRI and BamHI sites; (**b**). and rAAV-TK vector was constructed (**c**)

We designed a Recombinant AAV based vector (rAAV) (Fig. 1) which contains the Herpes Simplex Virus-1 (HSV-1) Thymidine Kinase (TK) gene under the control of the HIV-1 promoter LTR (rAAV-LTR-TK). AAV-DJ Helper Free Promoterless Expression System was purchased from CELL BIOLABS (Catalog number: VPK-411-DJ).

The complete sequence of the wild type LTR which contains the TAR structure (ClaI and EcoRI enzyme sites at the 5' and 3' end, respectively) was synthesized. The sequence was obtained from a 2nd generation lentivector; pLEX—jRED—TurboGFP; (Catalog number: OHS4736). The sequence was cloned into the PUC57 cloning vector, and subcloned into the above mentioned restriction enzyme sites of pAAV-MCS promoterless expression vector.

TK gene cloning was accomplished as follows. HSV-1 TK (Accession number: AB009254.2) was amplified by PCR from a PCDH-CMV-MCS-EF1-Puro (Catalog number: CD510B-1) plasmid carrying the coding region of TK, with primers containing EcoRI site at 5' and BamHI at 3' end. The primers sequences were:

TK Forward primer: 5' CAT CGA TGG AAT TCC TCC GTG TTT CAG TTA GCC 3'.

TK Reverse primer: 5' CG GGA TCC CAC CGA GCG ACC CTG 3'.

The PCR product was inserted downstream of the HIV-1 LTR which was cloned before into the pAAV-MCS promoterless expression vector, and the final vector was constructed (rAAV-LTR-TK).

#### Production of the recombinant AAV LTR-TK virus

One day before transfection, HEK-293 cells were cultured to achieve 70–80% confluence. Cotransfection of HEK-293 cells with rAAV-LTR-TK vector, pAAV-DJ and pHelper (with the ratio of 1:1:1 respectively) was achieved by using Calcium Phosphate transfection. 48–72 h after transfection rAAV-LTR-TK viruses were harvested by Freeze/thaw cycles based on the manufacturer instructions. rAAV LTR-TK crude lysate was used directly. rAAV LTR-TK aliquots were stored in −80 °C. After viral DNA extraction, virus titer was determined by qPCR.

### rAAV-LTR-TK transduction

CHME5/HIV − and CHEM5/HIV + cell lines cultured in DMEM, supplemented with 10% Fetal Calf serum and 1% penicillin/streptomycin, were plated on 24-well plates. 24 h later, cells were infected with rAAVs with a titer of 10,000 genome copies / cell (GC/cell). We checked the level of rAAV transduction in both CHME-HIV + and CHME-HIV- cells by qPCR. For this purpose, we performed qPCR to quantify the gene encoding TK which reflects the level of AAV transduced in both CHME5-HIV- and CHME5-HIV. The levels of AAV in both cell lines were comparable.

After 24 h of infection, cells were washed with PBS twice and treated with Romidepsin (Concentration: 0.0175 μM) to reactivate the latent virus. Next day, Ganciclovir (GCV) was added to the cells at final concentration of 2 mM and 24 h later, apoptosis assay was done according to “ab219918 Annexin V-iFluor 594 Apoptosis Detection Kit” (Abcam). MTT test for GCV was also performed according to Molecular Probes™ MTT (3-(4,5-Dimethylthiazol-2-yl)-2,5-Diphenyltetrazolium Bromide) ref: 10,133,722 Fisher Sci. The Percentage of cell reactivation and apoptosis was determined by flow cytometry. For each analysis, at least 5000 events were counted.

We also checked TK expression following Romidepsin treatment in CHME5-HIV + cells transduced with rAAV. We used relative RTqPCR methodology (method of the −2ΔΔ^CT^). Quantification was normalized with GAPDH.

### Statistical analysis

We performed at least 3 independent experiments for each condition. Data were presented as means and standard deviations (SD). Statistical analysis was performed by GraphPad Prism 9.0.0. Statistical significance was determined using “unpaired t-test with Welch’s correction” for comparison between two conditions that do not have equal SDs and “Ordinary one-way ANOVA” to compare 3 or more conditions. *p* value < 0.05 was considered statistically relevant and was indicated by stars (*p < 0.05, **p < 0.01, ***p < 0.001, ****p < 0.0001).

## Results

### The suicide gene strategy

**Fig. 2:**
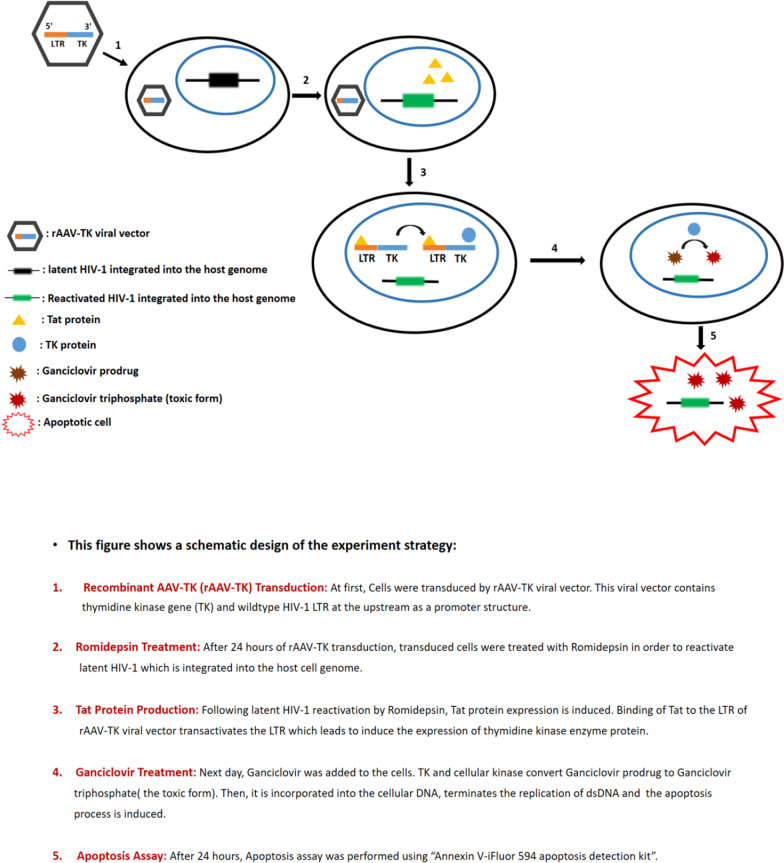
Schematic design of the experimental strategy

In the experiments recombinant AAV (rAAV) was used to infect CHME5/HIV + cells, a model of latently infected microglial cell line. The rAAV contained the gene coding for the herpes simplex (HS) thymidine kinase and transcription of the vector was driven by the 5 ‘Long Terminal Repeat (LTR) HIV-1 promoter. Cells expressing the herpes simplex (HS) thymidine kinase (Fig. 2 step 1) were treated with the HDAC inhibitor Romidepsin. In theory Romidepsin induces Tat protein expression by the reactivated virus (Fig. 2 step 2), which in turn activates the synthesis of thymidine kinase (TK) by binding on the LTR-TK promoter. Subsequently cells were treated with the prodrug Ganciclovir. It is awaited that in the presence of TK Ganciclovir is phosphorylated to Ganciclovir triphosphate (Fig. 2 step 3), a toxic form of the drug inducing apoptosis (Fig. 2 step 4). This strategy aims to reactivate specifically latently-infected cells. We expected that only reactivated cells are apoptotic when treated with Ganciclovir.

### Transduction of rAAV leads to apoptosis of CHME5/HIV + but not that of CHME5/HIV − cells

#### Negative control experiments

**Fig. 3:**
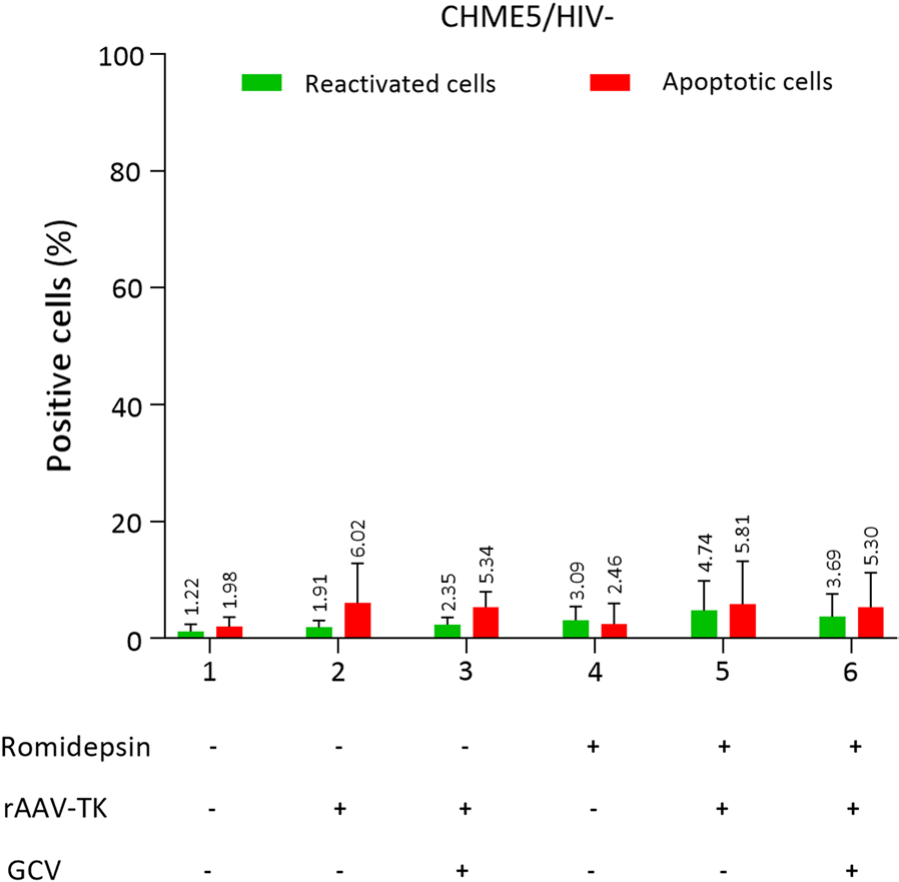
CHME5/HIV − cells were treated with Romidepsin, GCV and rAAV-TK viruses in different conditions. GFP + percentage represent the cells that are reactivated. RFP + percentage represents the cells that are apoptotic. The results were represented as mean and standard deviation of at least 3 independent experiments

In a first set of experiments, we used as control the non-latently-infected microglial cell line CHME5 (referred as CHME5/HIV −). The signal level of GFP reflects the percentage of reactivation of latently infected CHME5 cells (referred as CHME5/HIV +), while the signal level of RFP reflects the percentage of apoptotic cells. As expected, the non-treated and rAAV non-transduced CHME5/HIV − cells were negative for both GFP and RFP (Fig. 3 lane 1 and 2). We next checked whether various treatments by Romidepsin and Ganciclovir do not affect CHME5/HIV − cells transduced or not with rAAV. We showed that in cells transduced with rAAV but not treated with Romidepsin, the percentage of apoptotic cells in the presence or absence of Ganciclovir was low suggesting that the rAAV vector did not induce apoptosis of HIV- cells (Fig. 3 lanes 2 and 3). The percentage of reactivated cells (GFP +) and apoptotic cells (RFP +) that were not transduced by rAAV but treated with Romidepsin, were also very low supporting the idea that Romidepsin has no effect on its own (Fig. 3 lane 4). The slight increase of reactivated cells (GFP +) in the presence of Romidepsin or absence of Romidepsin (~ 3.5% and ~ 1.9% of GFP + , respectively) might reflect the autofluorescence of Romidepsin. In cells transduced with rAAV and treated with Romidepsin in the presence or absence of Ganciclovir, the signal levels of GFP and RFP (Fig. 3 lanes 5 and 6) were also very low, suggesting that Ganciclovir alone did not affect cell viability. The low level of reactivated CHME5/HIV − cells treated with a combination of drugs (Romidepsin and Ganciclovir) and transduced by rAAV (Fig. 3 lane 6) reflects the non-specific background luminosity. Of note, we might expect transcriptional activation of the TK gene driven by the LTR following Romidepsin treatment which could lead to unexpected apoptosis of non-reservoirs (CHME5 without HIV referred as CHME5/HIV −). However, we did not observe such an off-target effect with Romidepsin. Comparing CHME5/HIV − cells transduced by rAAV with and without Romidepsin treatment we notice that the level of apoptotic cells (RFP +) is low, which could reflect non- specific background luminosity (Fig. 3 lane 3 versus lane 6).

#### Experiments using the latently-infected microglial cell line CHME5/HIV + 

**Fig. 4:**
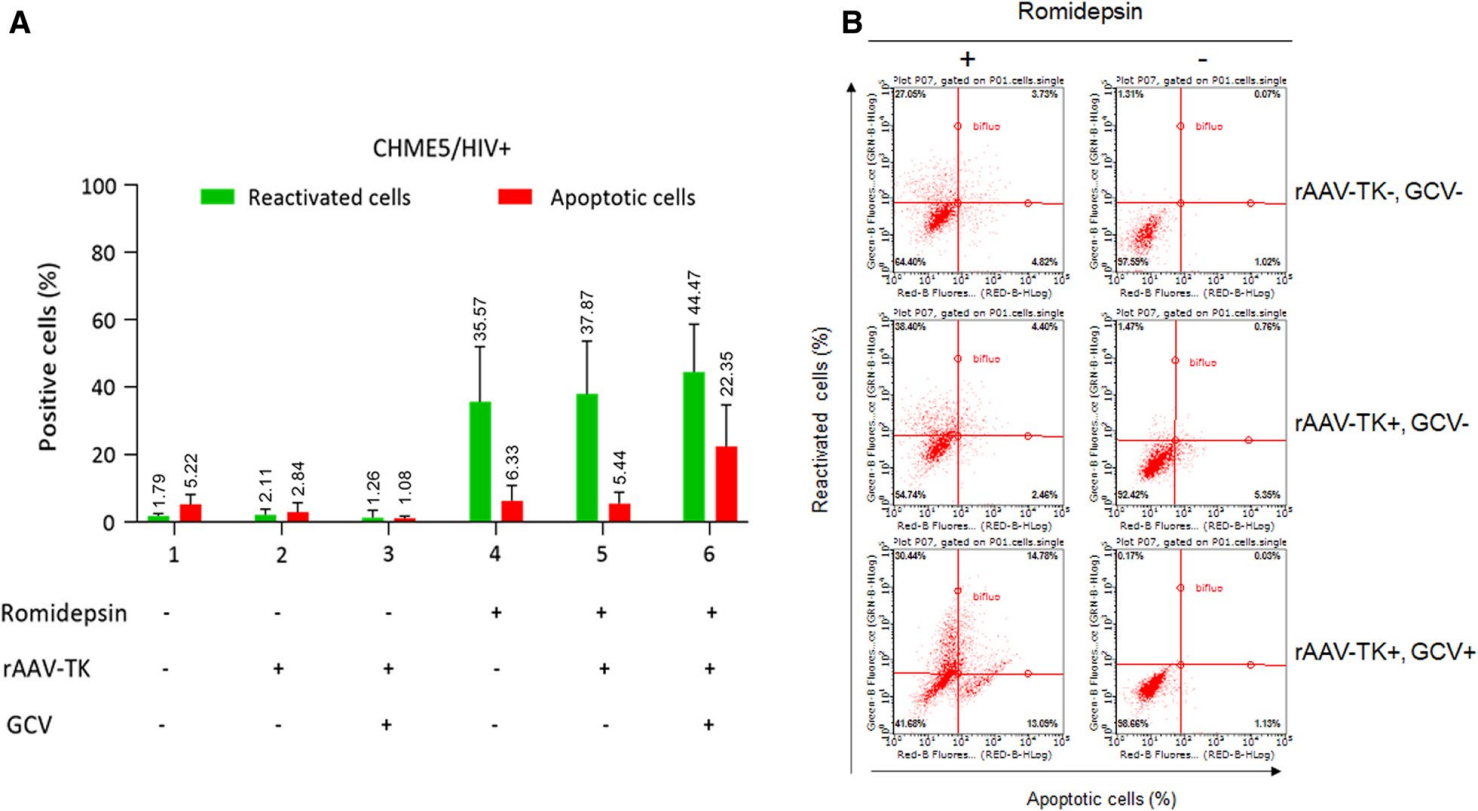
A-E CHME5/HIV + cells were treated with Romidepsin, GCV and rAAV-TK viruses in different conditions and latent virus reactivation and cell apoptosis was measured by flow cytometry. GFP + percentage represent the cells that are reactivated. RFP + percentage represents the cells that are apoptotic. The results of the histogram were represented as mean and standard deviation of at least 3 independent experiments. The dot plots is representative of one experiment

We next used the latently-infected microglial cell line CHME5/HIV + as a model and applied the same protocol as for the control CHME5/HIV − experiments (Fig. 4). We monitored the expression of GFP (Y axis) which reflects the percentage of reactivated CHME5/HIV + cells and the expression of RFP (X axis) which reflects the percentage of apoptotic cells. Representative dot plots of cell gates shows reactivated CHME5/HIV + cells (upper right and left quadrant) and apoptotic cells (upper and down right quadrant) (Fig. 4b). We visualized reactivated cells (GFP +) following Romidepsin treatment on the Y axis (compare right and left representation of the dot plots figure B). Interestingly, we only observe apoptotic cells (RFP +) among CHME5/HIV + cells transduced with rAAV-TK and treated with Romidepsin and Ganciclovir ((Fig. 4b compare the bottom right and left representations of the dot plots). We observed around 40% reactivation of CHME5/HIV + cells following Romidepsin treatment in the presence or absence of Ganciclovir (Fig. 4 lanes 4, 5 and 6). On the other hand, in the absence of Romidepsin, reactivation of CHME5/HIV + is low and comparable to the non-specific background observed with CHME5/HIV − cells (compare Fig. 4 lane 2 and 3 with Fig. 3 lane 2 and 3). Around 22% of the cells are apoptotic (RFP +) in the presence of Ganciclovir (Fig. 4 lane 6). In CHME5/HIV + cells transduced with rAAV-TK and treated with Ganciclovir we still observe some GFP + cells when treated with Romidepsin but subsequently they become RFP + that is apoptotic. The level of reactivation following Romidepsin treatment is comparable in CHME5/HIV + cells not transduced or transduced with rAAV (around 35 to 40%) (Fig. 4 lane 4 versus lanes 5 and 6). Interestingly, in cells not transduced with rAAV but treated with Romidepsin, the level of apoptotic cells (RFP +) was very low with a similar background level of fluorescence as observed in CHME5/HIV − cells (compare Fig. 4 lane 4 with Fig. 3 lane 4). Finally, CHME5/HIV + cells transduced with rAAV and treated with Ganciclovir needed to be reactivated by Romidepsin to generate fluorescence (GFP + and RFP +) (Fig. 4 lane 3 versus 6).

**Fig. 5:**
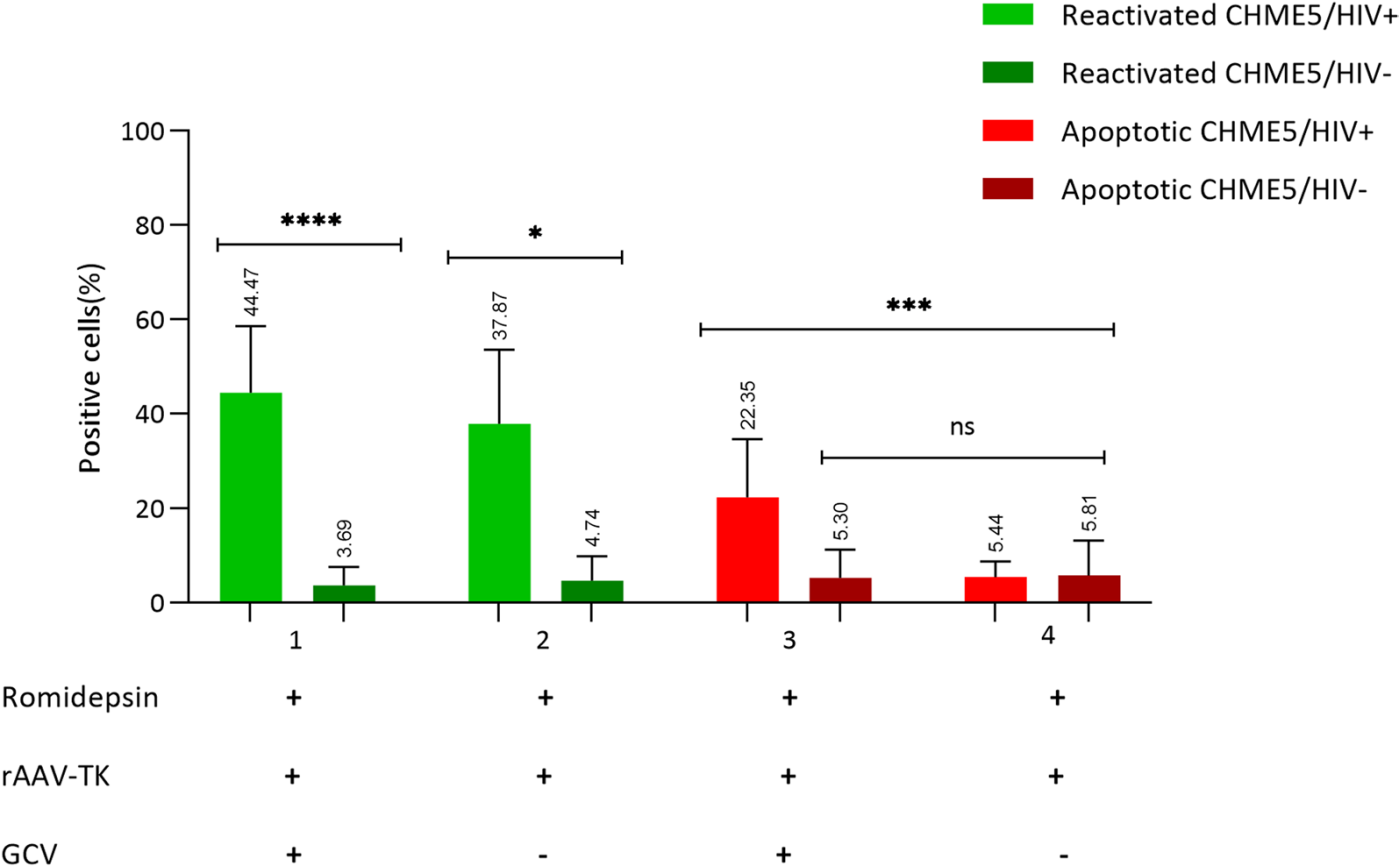
A comparison between CHME5/HIV + cells and CHME5/HIV − cells in different conditions, GFP + percentage represent the cells that are reactivated. RFP + percentage represents the cells that are apoptotic. Romidepsin (R), rAAV-TK (A) and GCV (G). The results were represented as mean and standard deviation of at least 3 independent experiments

A synthesis of our results is shown in Fig. 5.In the figure the effects of the various treatments on CHME5/HIV + and CHME5/HIV − cells are compared. The figure demonstrates well that cells reactivated by Romidepsin were exclusively CHME5/HIV + cells (GFP + lane 1 and 2) and only cells treated with Ganciclovir were apoptotic (RFP + lane 3 versus 4).

## Discussion

There is now numerous evidence suggesting that the major cellular reservoir of HIV-1 in the brain are microglial cells [7, 33]. These cells are believed to be the source of peripheral tissues reseedings and responsible for the emergence of drug resistance. Moreover, these lifelong reservoirs are known to be responsible for the development of HIV-1 associated neurocognitive diseases (HAND). Microglial cells differentiate from the myeloid precursors of the embryogenic yolk sac at a very early stage of embryogenesis [41, 42]. They colonize the entire brain parenchyma before the formation of the Brain Blood Barrier and form a slowly dividing population of cells in the brain [41]. In a recent study, the average age of microglial cells was evaluated to 4.2 years and the great majority of them were shown to be capable of cell division all along their life-time [43]. This means that infected microglial cells make brain infection permanent contrary to other infected cells found in the brain.

Targeting these cells located in anatomic and pharmacologic sanctuaries is however very challenging [7]. In the classic “Shock and Kill” strategy latently-infected T CD4 + cells are first reactivated by the Shock strategy and the newly formed viruses are eliminated by cART. On the other hand, reactivated HIV-1 positive cells are eliminated by the Kill strategy. A main backlash of the Kill strategy is that it is unable to eradicate all reservoirs despite latent cells becoming productive following reactivation. The failure is essentially due to the complexity of the Kill strategy, which does not allow direct elimination of reactivated cells. An original way to circumvent this problem is to use the suicide gene therapy. A major advantage of the strategy is that it eliminates reactivated cells directly and specifically by apoptosis. Thus in case of microglial cells neurotoxic viral proteins such as Tat and Vpr are not produced neither released. This could be a major improvement compared to the classical Kill strategy which has no effect on viral transcription even when enhanced cART is applied. Although much efforts have been employed to discover new molecules inhibiting the transcriptional step no drugs has been put on the market to date [4].

Here we have set up a gene suicide strategy using a latently infected microglial model. We used the potent HDAC inhibitor Romidepsin to reactivate latently-infected microglial cells. HDAC contributes to the transcriptional silencing of HIV-1 by inducing heterochromatin formation. First we showed that Romidepsin is able to reverse specifically heterochromatin silencing of the virus. Next we demonstrated that Ganciclovir treatment induces apoptosis of Romidepsin activated recombinant cells. Control cells, which were not transduced with rAAV were unaffected by treatments. Remarkably, only reactivated cells became apoptotic. These results strongly support the concept that the suicide gene strategy allows the specific elimination of latently infected cells.

It is noteworthy that Romidepsin treatment does not allow complete reactivation of latently infected cells. As in other strategies aiming to reactivate latent reservoirs, a combination of LRAs chosen from an existing pool of 160 LRA drugs should improve the level of reactivation [44, 45].

Although gene therapies show huge promises, they still need to be improved to achieve functional cure. Two main directions should be investigated: (i) designing efficient LRAs for the CNS and (ii) improving delivery of HIV drugs into the CNS and into the various CNS cells.

Several HDAC inhibitors (HDACi) have been tested in primary cells (astrocytes and macrophages) [46, 47]. Preliminary data showed that some LRAs including panobinostat [48] and JQ1 [49] are relatively nontoxic and are able to trigger HIV reactivation at a therapeutic concentration [47]. Bryostatin-1, an LRA drug which activates Protein Kinase C and crosses the blood brain barrier could be exploited in the suicide gene strategy especially if designed for microglial cells [50, 51].

Improvement of both the bioavailability and the bio-distribution of LRAs is also awaited with the development of carriers such as liposomes, dendrimers and micelles. Adeno-associated viruses are good candidates for delivering transgenes such as the LTR-TK transgene and/or drugs into the brain. Among the many serotypes tested AAV9 and AAV8 were shown to cross the BBB, which makes intravascular administration of the vectors feasible [52]. In primary human brain microvascular endothelial cells used as a model of human BBB, AAV9 was shown to cross the BBB and transduce brain cells following systemic administration [53]. rAAV9 was further tested in a clinical trial to treat neurological disorders [54]. However, gene transfer with rAAV9 was less effective in the adult than in the neonatal brain. A recent study found that systemic administration of an AAV8 mutant resulted in a more widespread brain gene transfer than that of rAAV9. Moreover, the study showed that BBB shuttle peptides improved AAV transduction in the brain [55].

## Conclusion

In conclusion, we provided proof of concept of the gene suicide strategy in this paper. Our results demonstrate that this strategy enables the eradication of latently-infected microglial cells. We expect that suicide gene therapy could be applied successfully in in vivo. This strategy could efficiently target latently-infected microglial cells in the brain, reactivating these cells by LRAs followed by their apoptosis. This approach thus prevents inflammation and the release of viral neurotoxic proteins which are secondary to reactivation. Indeed, the suicide gene approach aims to kill reactivated infected cells and simultaneously inhibit the release of new particles and viral products. The ultimate purge or at least considerable reduction of brain reservoirs, especially of microglial cells, is conceivable.

## Data Availability

Contact the corresponding authors Christian Schwartz or Mehrdad Ravanshad.

## Abbreviations

AAV: Adeno associated virus
BBB: Blood brain barrier
cART: Combination antiretroviral therapy
CNS: Central nervous system
CD4: Cluster of differentiation 4
CTLs: Cytotoxic T cells
CTIP2: COUP-Tf interacting protein 2
CCR5: Chemokine receptor type 5
CHME5/HIV−: Non-latently-infected microglial cell line
CHME5/HIV+: HIV-1 latently-infected microglial cell line
dCA: Didehydro-corticostatin
GCV: Ganciclovir
GFP: Green fluorescent protein
gp120: Glycoprotein 120
HAND: HIV-1 associated neurocognitive diseases
HIV-1: Human immunodeficiency virus 1
HDAC: Histone deacetylase
HSV: Herpes simplex virus
LRAs: Latency-reversing agents
LPAs: Latency-promoting agents
LSD1: Lysine-specific histone demethylase 1
LTR: Long terminal repeat
PKC: Protein kinase C
P-TEFb: Positive transcription elongation factor
RFP: Red fluorescent protein
SUV39H1: Suppressor of Variegation 3–9 Homolog 1
TK: Thymidine kinase
